# Chronic subdural hematoma—incidence, complications, and financial impact

**DOI:** 10.1007/s00701-020-04398-3

**Published:** 2020-06-10

**Authors:** Minna Rauhala, Pauli Helén, Heini Huhtala, Paula Heikkilä, Grant L. Iverson, Tero Niskakangas, Juha Öhman, Teemu M. Luoto

**Affiliations:** 1 Department of Neurosurgery, Tampere University Hospital and Tampere University, Tampere, Finland; 2 Faculty of Social Sciences, Biostatistics Group, Tampere University, Tampere, Finland; 3 Tampere Center for Child Health Research, Faculty of Medicine and Health Technology, Tampere University and University Hospital, Tampere, Finland; 4 Department of Physical Medicine and Rehabilitation, Harvard Medical School; Spaulding Rehabilitation Hospital and Spaulding Research Institute; & Home Base, A Red Sox Foundation and Massachusetts General Hospital Program, Boston, Massachusetts USA; 5 Faculty of Medicine and Life Sciences, Tampere University, Tampere, Finland

**Keywords:** Chronic subdural hematoma, Recurrence, Follow-up, Health care costs, Excess mortality, Causes of death, Survival

## Abstract

**Objective:**

To examine the population-based incidence, complications, and total, direct hospital costs of chronic subdural hematoma (CSDH) treatment in a neurosurgical clinic during a 26-year period. The aim was also to estimate the necessity of planned postoperative follow-up computed tomography (CT).

**Methods:**

A retrospective cohort (1990–2015) of adult patients living in Pirkanmaa, Finland, with a CSDH was identified using ICD codes and verified by medical records (*n* = 1148, median age = 76 years, men = 65%). Data collection was performed from medical records. To estimate the total, direct hospital costs, all costs from hospital admission until the last neurosurgical follow-up visit were calculated. All patients were followed until death or the end of 2017. The annual number of inhabitants in the Pirkanmaa Region was obtained from the Statistics Finland (Helsinki, Finland).

**Results:**

The incidence of CSDH among the population 80 years or older has increased among both operatively (from 36.6 to 91/100,000/year) and non-operatively (from 4.7 to 36.9/100,000/year) treated cases. Eighty-five percent (*n* = 978) underwent surgery. Routine 4–6 weeks’ postoperative follow-up CT increased the number of re-operations by 18% (*n* = 49). Most of the re-operations (92%) took place within 2 months from the primary operation. Patients undergoing re-operations suffered more often from seizures (10%, *n* = 28 vs 3.9%, *n* = 27; *p* < 0.001), empyema (4.3%, *n* = 12 vs 1.1%, *n* = 8; *p* = 0.002), and pneumonia (4.7%, *n* = 13 vs 1.4%, *n* = 12; *p* = 0.008) compared with patients with no recurrence. The treatment cost for recurrent CSDHs was 132% higher than the treatment cost of non-recurrent CSDHs, most likely because of longer hospital stay for re-admissions and more frequent outpatient follow-up with CT. The oldest group of patients, 80 years or older, was not more expensive than the others, nor did this group have more frequent complications, besides pneumonia.

**Conclusions:**

Based on our population-based study, the number of CSDH patients has increased markedly during the study period (1990–2015). Reducing recurrences is crucial for reducing both complications and costs. Greater age was not associated with greater hospital costs related to CSDH. A 2-month follow-up period after CSDH seems sufficient for most, and CT controls are advocated only for symptomatic patients.

## Introduction

Chronic subdural hematoma (CSDH) is a common disease in neurosurgical practice among elderly patients and it is associated with substantial morbidity and mortality [5, 10, 25, 27, 41]. The incidence of CSDH has increased during the last decades [1, 4, 14, 18]. We have previously published the epidemiological findings of our Finnish CSDH cohort (1990–2015), in which the overall incidence doubled from 8.2 to 17.6/100,000/year [33], and nearly tripled among the population 80 years or older. The global population of people aged 80 and older is expected to more than triple between 2015 and 2050 [16]. Consequently, there is a growing healthcare burden related to CSDH. Only a few studies have described the financial impact of CSDH [12, 13, 35].

Surgical treatment is recommended in CSDH patients with neurological symptoms, and the preferred surgical technique is burr-hole drainage [26, 37]. Recurrence is common, ranging from approximately 5 to 30%, and a reduced recurrence rate is observed with external subdural drains [22, 31, 40]. Routine postoperative CT can potentially detect recurrent CSDH before clinical deterioration occurs [9]. A concern has been raised, however, that unnecessary revision surgery and increased costs may outweigh this benefit [30]. The usefulness of routine follow-up CT to predict symptomatic recurrence is questionable [35]. There are no guidelines on how, or for how long, CSDH patients should be followed. In our neurosurgical unit, an outpatient clinic follow-up visit with a head CT 4 to 6 weeks after the operation is a routine.

The objective of this study was to examine the incidence, complications, and total, direct hospital costs of CSDH treatment from hospital admission until the last follow-up visit in a neurosurgical clinic during a 26-year study period. The aim was also to evaluate the necessity of pre-scheduled routine follow-up CT after CSDH. A large unselected, population-based CSDH patient cohort from 1990 to 2015 was analyzed. We hypothesized that costs are high and increasing because the incidence of CSDH is increasing, re-operations are frequent, and the duration of overall treatment (including follow-up visits) is long. We also hypothesized that there would be a substantial percentage of patients that would have asymptomatic post-operative recurrence of their hematomas visible on follow-up CT.

## Methods

### Material and ethical aspects

The study was conducted in the Department of Neurosurgery at the Tampere University Hospital (Tampere, Finland). Patients included were (1) residents in the Pirkanmaa Region, (2) aged 18 years or over with no upper limit, and (3) diagnosed with CSDH between 1990 and 2015. The cases were retrospectively identified using the hospital’s patient administrative databases, including International Classification of Diseases (ICD) codes for traumatic and non-traumatic subdural hematomas (SDHs; ICD-10 codes: S06.5 and I62.0; ICD-9 codes: 432.1, 852.2 and 852.3). Verified cases were classified by SDH type (acute, subacute, chronic, and hygroma) by reviewing all the medical records. Exclusion criteria were acute or subacute SDH (< 3 weeks after head trauma), hygroma (a collection of subdural cerebrospinal fluid without any signs of blood), and any form of intracranial surgery within 12 months preceding the CSDH diagnosis.

The Pirkanmaa Region is a geographically well-defined area with both rural and urban areas that holds one of Finland’s five neurosurgical departments (Department of Neurosurgery, Tampere University Hospital, Tampere, Finland). All neurosurgical cases from this area are referred to the Tampere University Hospital, with a catchment population of one million inhabitants. To investigate the population-based burden of CSDH, we collected data on patients with CSDHs who were residents of the Pirkanmaa Region. Over 9% of the Finnish population lives in the Pirkanmaa Region. The population increased from 427,223 in 1990 to 506,114 in 2015. The population over 80 years old almost doubled from 13,565 to 26,417 during the study period. The annual number of inhabitants in the Pirkanmaa Region was obtained from the Statistics Finland (Helsinki, Finland).

The study was approved by the Ethics Committee of the Pirkanmaa Hospital District, Tampere, Finland (ethical code: R12082). All data was collected retrospectively without contacting the patients; therefore no, written informed consent was obtained or required.

### Data collection

A detailed and structured data collection was performed from medical records. Results from imaging reports were coded; CT scans or MR images were not examined directly. Patients were stratified into three groups according to age: (i) 18–59 years, (ii) 60–79 years, and (iii) ≥ 80 years. The study period was divided into five time periods: (i) 1990–1995, (ii) 1996–2000, (iii) 2001–2005, (iv) 2006–2010, and (v) 2011–2015. CSDH recurrence was defined as an ipsilateral hematoma needing re-operation within 2 years of the original operation. All patients were followed until death or the end of year 2017.

### Cost data

To estimate the direct, total hospital costs from admission until the last follow-up visit, we calculated all costs for the whole treatment period, including operations and number of days spent in the neurosurgical ward, recovery room or ICU, emergency department visits related to the CSDH, laboratory and radiologic costs, and follow-up visits. The possible period of rehabilitation following treatment in the neurosurgical ward was not included the financial calculations. The unit costs (presented in Table 3) were then multiplied by the number of cost factors of each patient. Because of the long study period, it was not possible to get all of the individual patient’s costs directly from our hospitals’ invoicing department. Instead, all costs were calculated from the latest 2018–2019 data from hospital administration and catalogues for in-hospital use.

When comparing the results from earlier studies, costs were first adjusted to year 2018 value by consumer price index of the relevant country [38, 39], and then converted to FIN EUR using the latest year 2018 Purchasing Power Parities (PPP) of that country. PPP tries to equalize the purchasing power of different currencies, by eliminating the differences in price levels between countries.

### Statistical analyses

SPSS (IBM SPSS Statistics for Windows, Version 25.0, Armonk, NY, USA) was used for data analyses. Descriptive statistics [frequency (*n*), percentage, median, interquartile range, range] were used to describe variable and subgroup characteristics. The chi-square test was used to compare differences between groups. The statistical significance level was set at *p* < 0.05. A Kaplan–Meier analysis was used for the time of first surgery to recurrence, and the hazard ratio was computed based on a Cox regression. Observations for event-free patients were censored at the time of death.

## Results

### Characteristics

**Fig. 1:**
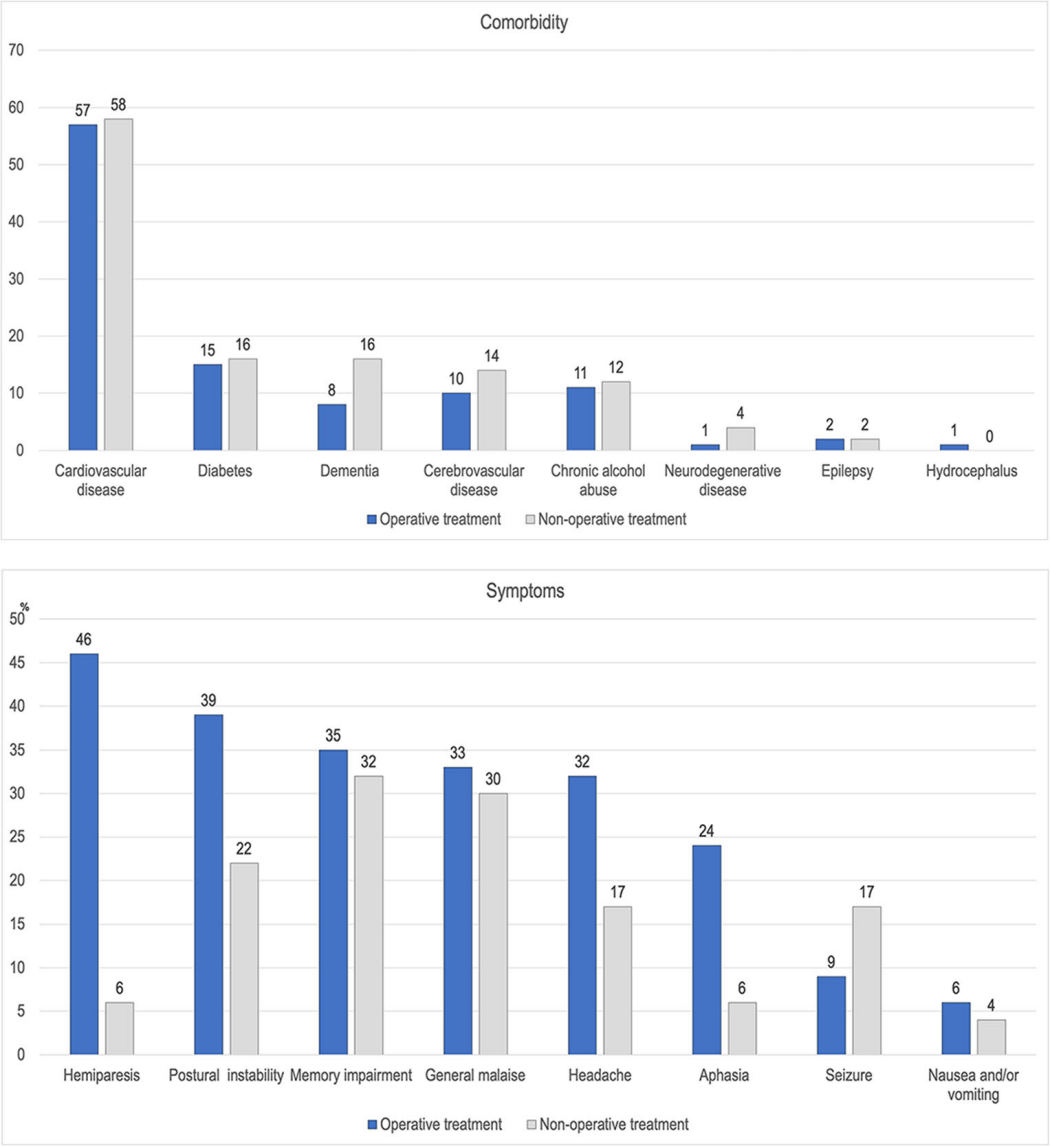
Comorbidities and symptoms of patients with chronic subdural hematoma stratified by treatment group

A total of 1133 unique patients with CSDH were identified. Patients with CSDH were considered new cases if 2 years had elapsed following primary treatment or if they had a new contralateral hematoma (*n* = 15). During the study period, 14 patients (1.2%) underwent new contralateral CSDH evacuation and only one patient (0.09%) needed operative treatment for the same sided CSDH after 5 years of index CSDH. Therefore, the total number of cases was 1148, and 748 (65%) were men. The median age for CSDH diagnosis was 76 years, increasing from 73 to 79 years during the 26-year period. Mortality was 3.4% in 30 days and 14% in 1 year. The characteristics of the whole sample and treatment subgroups are presented in Table 1 and Fig. 1. We have previously published the details of CSDH patients from this same patient cohort stratified by gender, age groups, and time periods [33].

**Table 1:** Characteristics of all patients with chronic subdural hematoma in Pirkanmaa that were treated at the Tampere University Hospital between 1990-2015.

	Total sample n = 1148		Non-operative treatment *n* = 170		Operative treatment *n* = 978		*p* value
	*n*	%	*n*	%	*n*	%	
Age, median, IQR (years)	76	67–83	79	68–86	76	66–82	0.005
Men	748	65.2	104	61.2	644	65.8	0.24
Traumatic etiology	679	59.1	109	64.1	570	58.3	0.15
Comorbidity	880	76.7	132	77.6	748	76.5	0.74
Chronic alcohol abuse	126	11.0	20	11.8	106	10.8	0.72
Medication							0.63
Antiplatelet	270	23.5	36	21.2	234	23.9	0.34
Warfarin	191	16.6	27	15.9	164	16.8	0.57
Warfarin AND antiplatelet	23	2.0	2	1.2	21	2.1	0.36
Neurological deficit (hemiparesis or dysphasia)	573	49.9	18	10.6	555	56.7	< 0.001
Admission mRS 0–3	585	51.0	122	71.8	463	47.3	< 0.001
Hematoma characteristics							
Left sided	478	41.6	71	41.8	407	41.6	0.67
Bilateral	257	22.4	34	20.0	223	22.8	0.42
Mortality*							
30 days	38	3.4	13	7.7	25	2.6	0.001
6 months	108	9.5	26	15.4	82	8.5	0.005
1 year	155	13.7	35	20.7	120	12.4	0.004
2 years	254	22.4	54	32.0	200	20.7	0.001

*IQR* interquartile range, *mRS* modified Rankin scale

^*^Patients with multiple CSDH episodes were excluded (*n* = 15)

### Incidence of CSDH stratified by treatment groups

**Fig. 2:**
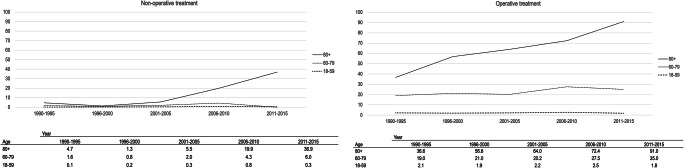
Incidence (*n*/100,000) of chronic subdural hematoma stratified by treatment group in different age groups during the study period between 1990–2015 in Pirkanmaa, Finland

From the time period of 1990–1995 to 2011–2015, the overall incidence of operatively treated CSDH in adults almost doubled from 7.2 to 13.4/100,000/year. The incidence remained quite stable among those under the age of 70 but increased 2.5 times for ≥ 80-year olds from 36.6 to 91/100,000/year. Prior to the 2001–2005 time period, only a small number of non-operatively treated patients were diagnosed. The incidence of non-operatively treated CSDH for those 80 years and older increased from the beginning of the millennium reaching 36.9/100,000/year (2011–2015). The incidence rates stratified by treatment groups are presented in Fig. 2.

### Non-operatively treated patients

Fifteen percent of cases (*n* = 170) were treated non-operatively. For most cases, the reason for non-operative treatment was that the CSDH did not cause significant neurological signs or symptoms. A very small number of patients were not offered surgery because they presented in a moribund state (*n* = 7). Non-operative treatment included discontinuation of possible antithrombotic medication, active mobilization, and follow-up CT scans (routinely or for emerging new symptoms). The median number of CT scans performed was 1.5 (min–max = 1–5). The median number of outpatient clinic follow-up visits was 0 (min–max = 0–4).

### Operatively treated patients

For the total sample, 978 (85%) were treated operatively. Median time from CT to surgery was 0 days, and 588 of patients (60%) were operated on the same day, 294 (30%) on day 1, and 50 (5%) on day 2 from diagnosis. Only 5% of patients were operated on day 3 or more from diagnosis. There were 53 patients (5%) who were first treated non-operatively but then underwent surgery when the CSDH increased in size (median time from first CT to surgery was 24 days). The operative treatment strategy has remained practically unchanged during the 26-year study period. The only evolution in the treatment has been the more frequent use of subdural drains during the last years of the study period. Most operations were performed under local anesthesia (*n* = 839; 86%) via one burr hole, and the hematoma was evacuated through irrigation. A subdural drain was inserted in 59 patients (6%). The drain was kept below the head level with no suction for 24–48 h. Only one patient underwent craniotomy as the primary surgery. The patients were actively mobilized directly after the operation. Antiepileptic drugs were not prescribed routinely. Most patients (72%) were seen in an outpatient clinic and underwent follow-up CT 4 to 6 weeks after the operation. In the case of residual hematoma needing no re-operation, patients were followed monthly until the hematoma resolved significantly. The median number of CT scans performed was 3 (min–max = 1–13), and the total number was 3043. The median number of outpatient follow-up visits was 1 (min–max = 0–11), and the total number was 1463.

### Recurrence

A recurrent hematoma was treated surgically in 278 cases (28%, median age 76 years). The recurrent hematoma was symptomatic in 229 (82%) of the cases, and operated because follow-up CT revealed a large CSDH in 49 of patients (18%). Twenty-two patients (8% of the recurrences) underwent a second surgery during the primary admission. A re-operation was done on 114 symptomatic patients (41% of the recurrences) before their scheduled outpatient clinic visit. The first scheduled outpatient visit with CT led to a re-operation in 108 patients (39% of the recurrences). Of these 108 patients, 70 (65%) had symptoms and 38 (35%) were symptom free. The median time to recurrence was 25 days (min–max = 0–304 days, IQR = 14–35). Most of the recurrences were treated operatively within 30 days (63%) or within 2 months (92%) after the primary operation. Only two patients underwent surgery after 6 months (285 days and 304 days) from their primary operation.

**Fig. 3:**
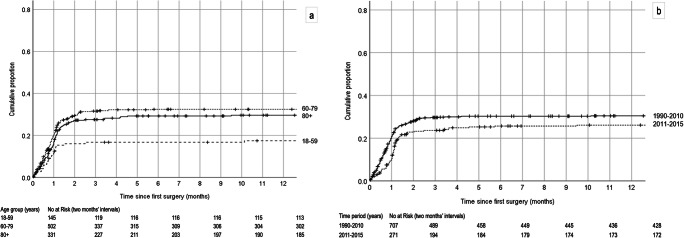
Cumulative proportion of recurrences shown as Kaplan–Meier analysis in different age groups (**a**) and time periods (**b**). Observations for event-free patients were censored at the time of death

The cumulative proportion of recurrences is shown in Fig. 3. The differences between the age group of 18–59 years, and both the age groups of 60–79 years (HR = 2.02; 95% CI = 1.32–3.08, *p* = 0.001) and ≥ 80 years (HR = 1.79; 95% CI = 1.15–2.77, *p* = 0.01), were significant. The recurrence rate among patients treated with drains was significantly lower than for patients with no drain (17% vs 29%, *p* = 0.04). Almost all patients with a drain (48/59; but still only 18% of all surgically treated patients) were from the last study period (2011–2015). During this period, the recurrence rate was 25%, which was non-significantly lower than the rate of previous years combined (HR = 0.79; 95% CI = 0.60–1.04, *p* = 0.10).

### Complications related to operative treatment of CSDH

The most common complication was a seizure occurring in 4.8% of the total sample of patients undergoing surgery. Acute intracranial hemorrhage was rare; there were 11 cases of acute subdural hematoma (1.1%) and 6 cases of intracerebral hematoma (0.6%). Nine (0.9%) out of these 17 required emergency craniotomies, and two (0.2%) patients died. Postoperative infection at the site of surgery was diagnosed in 29 (3.0%), and 20 (2.0%) patients underwent a second surgery because of an empyema.

The only complication with a significant difference between the age groups was pneumonia occurring more often among the oldest patients (≥ 80-years, *p* = 0.02). Patients undergoing a second surgery suffered more often from seizures (10%, *n* = 28 vs 3.9%, *n* = 27; *p* < 0.001), empyema (4.3%, *n* = 12 vs 1.1%, *n* = 8; *p* = 0.002), and pneumonia (4.7%, *n* = 13 vs 1.4%, *n* = 12; *p* = 0.008) compared with patients with no recurrence. The complications are shown in Table 2.

**Table 2:** Morbidity, perioperative complications, and mortality in patients with an operatively treated chronic subdural hematoma

	Total *n* = 978				Age group					Recurrence				
			18–59 *n* = 145		60–79 *n* = 502		≥ 80 *n* = 331		*p* value	No *n* = 700		Yes *n* = 278		*p* value
	*n*	%	*n*	%	*n*	%	*n*	%		*n*	%	*n*	%	
Comorbidity	748	76.5	74	51.0	377	75.1	297	89.7	< 0.001	534	76.3	214	77.0	0.82
Complications														
Seizures	55	5.6	5	3.4	33	6.6	17	5.1	0.32	27	3.9	28	10.1	< 0.001
Acute subdural hematoma	11	1.1	1	0.6	8	1.4	2	0.5	0.30	6	0.9	5	1.8	0.21
Intracerebral hemorrhage	6	0.6	0	0	5	0.9	1	0.2	0.24	3	0.4	3	1.1	0.24
Cerebrovascular infarction	6	0.6	1	0.6	3	0.5	2	0.5	1.0	3	0.4	3	1.1	0.24
Surgical site infection	29	3.0	1	0.7	20	4.0	8	2.4	0.92	15	2.1	14	5.0	0.016
Empyema	20	2.0	1	0.7	16	3.2	3	0.9	0.05	8	1.1	12	4.3	0.002
Pulmonary embolus	2	0.2	0	0	1	0.2	1	0.3	0.80	1	0.1	1	0.4	0.50
Pneumonia	25	2.6	0	0	11	2.2	14	4.2	0.020	12	1.7	13	4.7	0.008
Discharge to home	409	41.8	93	64.1	253	50.4	63	19.0	< 0.001	291	41.6	118	42.4	0.80
Mortality														
30 days	25	2.6	2	1.4	12	2.4	11	3.4	0.43	23	3.3	2	0.7	0.022
6 months	82	8.5	4	2.8	38	7.6	40	12.3	0.002	66	9.5	16	5.9	0.06
1 year	120	12.4	6	4.2	50	10.1	64	19.8	< 0.001	92	13.3	28	10.3	0.20
2 years	200	20.7	14	9.7	84	16.9	102	31.5	< 0.001	145	21.0	55	20.1	0.77

### Length of hospital stay and discharge

Most non-operatively treated patients were not treated in our neurosurgical ward, so the median length of hospital stay was 0 days (min–max = 0–4 days). The median length of hospital stay for surgically treated patients was 3 days (min–max = 1–33 days), and 4 days (min–max = 1–46 days) during the re-admission for patients with a recurrent hematoma. The number of days the surgically treated patients spent in our neurosurgical ward decreased during the study period from 5 days (1990–1995) to 3 days (2011–2015). The proportion of patients discharged home did not change significantly; it was 49% in 1990–1995 and 43% in 2011–2015 (*p* = 0.25). The rate of home discharge was as follows: 18–59 years, 64%; 60–79 years, 50%, and ≥ 80 years, and 19% (*p* < 0.001).

### Hospital costs

The mean total cost from the first hospital admission until the last follow-up visit per patient treated surgically was 5250 € (median 3810; €; IQR = 2930–5900 €) (Table 3). It was 3820 € (median 3370 €; IQR = 2870–4100 €) per patient for those with no recurrence, and 8850 € (median 7110 €; IQR = 5840–9820 €) per patient for those with recurrence. The difference in mean hospital costs of 5030 € reflects 132% higher costs for the patients with recurrence. The mean cost for patients with drains was 4100 € (median 3370 €; IQR = 2930–6050 €) and for those with no drains 5320 € (median 3930 €; IQR = 2610–5500 €). The difference in mean treatment costs of 1220 € reflects 30% higher costs for patients with no drains compared with patients with drains.

**Table 3:** Costs related to treatment of chronic subdural hematoma in Tampere University Hospital during the study period between 1990-2015

	Non-operative treatment	Operative treatment	Cost á
No of operations	0	1 (1–8)	982 €/h*
Recovery room stay 2 h in the case of general anesthesia	NA	0 (0–3)	290 €/2 h
ICU stay in the case of craniotomy	NA	0 (0–2)	1376 €/day
Hospital stay in neurosurgical unit during primary admission, days	0 (0–4)	3 (1–33)	440 €/day
IQR	0	3–5	
Hospital stay in neurosurgical clinic during re-admission, days	NA	4 (1–46)	440 €/day
IQR	NA	3–8	
CT scans, median, *n*	1.5 (1–5)	3 (1–13)	147 €
Laboratory tests taken at the time of diagnosis, re-admission and/or complication	1	1 (1–10)	50 €
Emergency department visits, n**	1 (1–2)	1 (1–5)	111 €
Outpatient follow-up visits, *n*	0 (0–4)	1 (0–11)	174 €
Costs in Euros per patient, min–max	310–2,710	2,170–33,420	
Mean	580	5,250	
Median	455	3,810	
IQR	310–630	2,930–5,900	
No recurrence	NA	2,170–28,980	
Mean	NA	3,820	
Median	NA	3,370	
IQR	NA	2,870–4,100	
Recurrence	NA	3,640–33,420	
Mean	NA	8,850	
Median	NA	7,110	
IQR	NA	5,840–9,820	

Data are shown as median (min–max); *IQR* interquartile range; *NA* not applicable

*The mean operation theatre time for burr hole trephination of CSDH was 1 h, and for craniotomy 3 h

**Patients were assumed to be diagnosed in emergency department

Among surgically treated patients, the 60–79 years old age group had the greatest costs; the mean cost was 5710 € (median 4050 €; IQR = 3130–6570 €), while the cost for those in the 18–59 years group (mean 4640 €; median 3690 €; IQR = 2930–5060 €), and over 80-year old group (mean 4810 €; median 3660 €; IQR = 2930–5600 €) were similar. The mean hospital cost per non-operatively treated patient was 580 € (median 455 €; IQR = 310–630 €).

**Fig. 4:**
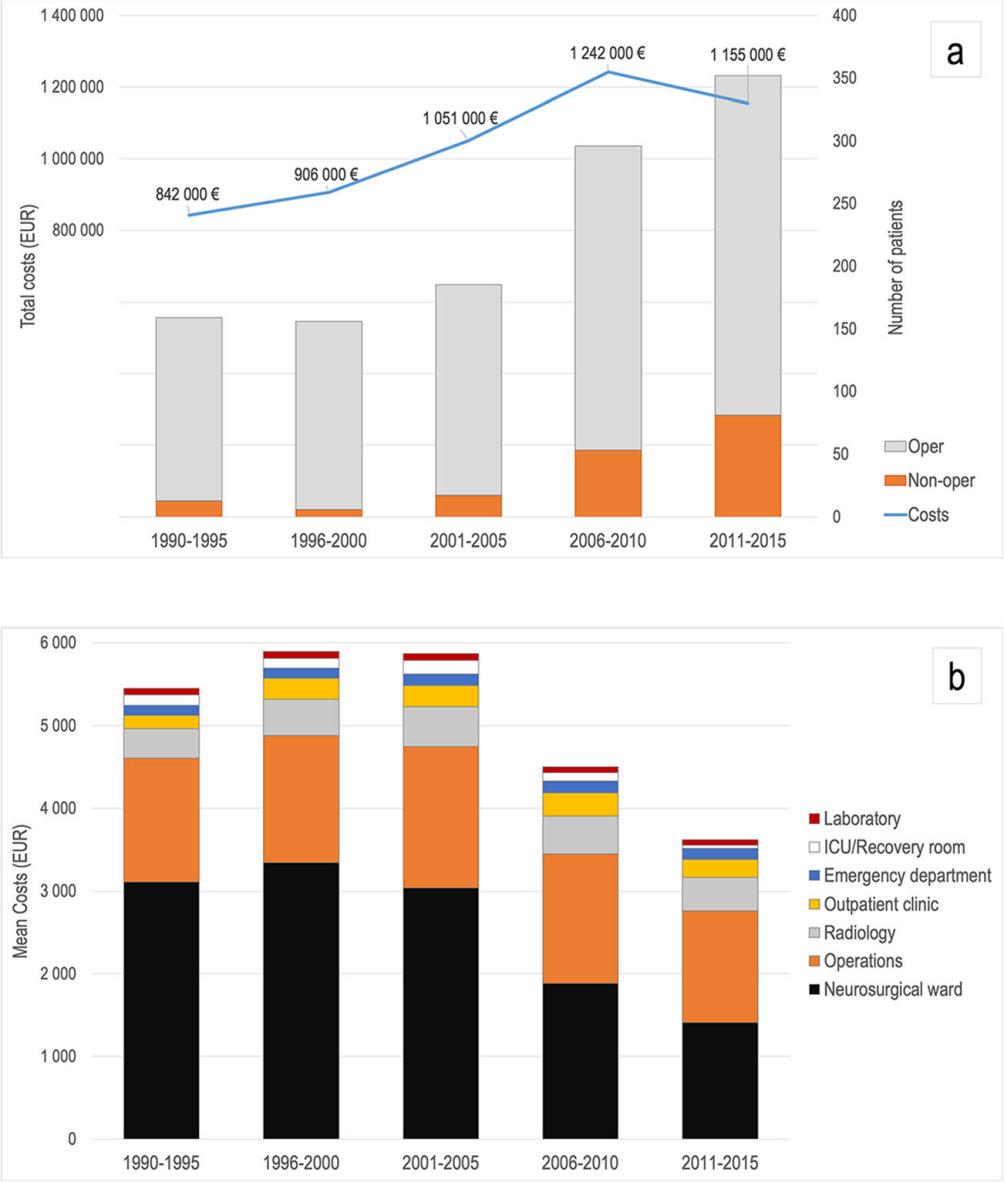
Direct costs of chronic subdural hematoma in Tampere University Hospital. Total hospital costs per 5-year time periods, and the number of operatively and non-operatively treated patients (**a**). A breakdown of mean hospital costs per patient stratified by time groups (**b**). The analysed CSDH patients living in Pirkanmaa Region accounted approximately half of all the CSDH patients treated in Tampere University Hospital with a catchment population of one million

The length of hospital stay is a large contributor to total costs. However, during the last 10 years (2006–2015), while the duration of hospital treatment has decreased, the cost of operative treatment has become approximately equal with the hospital stay. The total, direct hospital costs for CSDH are presented in Table 3 and Fig. 4, and a breakdown of mean hospital costs per patient stratified by time period is presented in Fig. 4.

The mean cost per patient was greatest during 1996–2000, when it was 5840 € (median 4040 €; IQR = 3250–6730 €). The greatest total hospital costs 1,242,000 € were during 2006–2010. The period with the lowest costs per patient was 2011–2015 (mean 3310 €; median 2930 €; IQR = 2170–4170 €). Because of this, even though the number of patients has increased, the total costs have decreased during the more recent years of the study period. Considering total hospital costs, the lowest cost period was 1990–1995, and the total costs were 842,000 €.

## Discussion

### Summary of the key findings

During the study period from 1990 to 2015, the incidence of CSDH among those 80 years and older has increased in both operatively (from 36.6 to 91/100,000/year) and non-operatively (from 4.7 to 36.9/100,000/year) treated patients. Patients prescribed routine post-operative head CT scans were more likely to undergo a second surgery, and 18% of those with a second surgery were asymptomatic during the follow-up visit in which the CT was conducted. Most of the second surgeries took place within 30 days (63%) or 2 months (92%) from the primary operation. Those undergoing a second surgery suffered more often from seizures (10% vs 3.1%), empyema (4.3% vs 0.9%), and pneumonia (4.7% vs 1.4%). The treatment cost for recurrent CSDHs was 132% higher than the treatment cost of non-recurrent CSDHs, most likely because of longer hospital stay for re-admissions and more frequent outpatient follow-up with CT. The costs were 30% higher for patients with no drain compared with patients with a drain, perhaps due in large part to more frequent recurrences needing second surgeries.

The mean cost per patient was greatest for the 60–79-year old age group, in large part because they were more likely to undergo a second surgery than patients in younger and older groups. The oldest group, 80 years and older, did not have greater costs than the others, nor did patients in this group have more complications, besides pneumonia. The mean cost per patient has decreased over the past two time periods (i.e., 2006–2015). This is explained mostly by reduced hospital stay and fewer recurrences requiring surgery. There were also more frequently diagnosed non-operatively treated patients, but their share of the costs was modest. The total costs increased through the 2006–2010 period, but then decreased during the 2011–2015 period, despite greater numbers of patients being treated. This relates to the decrease in the mean cost per patient.

### Comparison of the current findings to prior literature

The incidence of CSDH has increased among the elderly during the last decades [1, 4, 14, 18, 33]. The reasons for this include age-related general brain atrophy [23, 42], risk for multiple falls [15, 19, 21], and the frequent use of antithrombotic medication [7, 8, 14, 29]. In addition, improved awareness of CSDH among the medical profession and the wide availability of CT scanners have been proposed to have an influence [2, 36]. The availability of CT likely contributes to the increase in non-operatively treated patients; asymptomatic CSDHs are found when the threshold for ordering imaging is low. There are no previous reports separating the incidence between the operatively and non-operatively treated patients.

Routine follow-up head CT reveals large recurring hematomas in some clinically asymptomatic patients, leading to the decision for a second surgery. In a Danish retrospective study of 202 patients with CSDHs, recurrence of neurological symptoms preceded the planned postoperative follow-up CT (4 to 6 weeks after primary surgery) in all patients undergoing a second surgery [30]. The Swiss TOSCAN trial with 361 randomized patients showed that routine CT scans did not improve clinical outcome but led to increased costs [35].

Our data support some previous findings. Our recurrence rate (*n* = 278/978; 28%), is toward the high end of what is reported in the literature [22, 31, 40]. Of the total sample of 978, 5% (*n* = 49) of the patients undergoing a second surgery were asymptomatic. Considering the 278 who had a recurrence, 18% were asymptomatic. Correspondingly, asymptomatic patients were operated on based on CT findings in the TOSCAN trial [35]. In the TOSCAN study, 27% of the CSDH patients with follow-up CT underwent a second surgery vs 19% with no prescribed CT. In our study, however, during the first follow-up visit, 70 patients of 108 (65%) with recurrence visible on CT were slightly symptomatic, but waited for the scheduled outpatient clinic visit. In addition, the use of subdural drains during the study period was uncommon (6%). Insertion of an external drain after evacuation of CSDH decreases the rate of recurrence in most of reported series by up to 50% [22, 31, 40].

The median time to re-operation, 25 days, is in line with previous studies. Mori et al. [28] reported a median time to re-operation of 24.5 days, Lutz et al. [24] 22.5 days, Pedersen et al. [30] 22 days, although Ridwan et al. [34] only 17 days. In our study, most of the second surgeries took place within 30 days (63%) or 2 months (92%) from the primary operation. Results from the TOSCAN trial were similar (68% within 30 days and 93% within 2 months) [35]. In addition, in a German study of 208 patients with a recurrence rate of 18%, the majority (92%) of recurrences occurred within 60 days [34].

Many patients with CSDHs are on antithrombotic medication, and this medication is paused at the time of diagnosis. The post-CSDH resumption of these drugs is not straightforward [32]. Nine of ten recurrences occur within 60 days. This information may help inform when to resume antithrombotics when needed, even with no follow-up CT. Notably, early resumption has also been advocated [32], and not all the patients can wait for 2 months due to a high risk of thromboembolic events. Further studies are warranted to investigate if early resumption of antithrombotics is safe without prescribing follow-up CT. In addition, there is a medico-legal issue of permission to drive. For these reasons, in selected cases, a planned follow-up CT may be necessary. However, it seems that in the majority of the cases, clinical follow-up and CT only for symptomatic patients, is just as good. When clinically indicated, a 2-month follow-up period after CSDH is likely sufficient for most asymptomatic patients.

### Cost comparison with previous CSDH studies

The literature on CSDH-related costs is scarce. In the TOSCAN trial (conducted June 2012–August 2016), the mean cost per patient from hospital admission until the last follow-up visit was 21,298 CHF (15,927 €) in the CT-arm and 18,047 CHF (13,497 €) in the no-CT-arm, the difference being 18% [35]. Median length of hospital stay was 6 days. The investigators pointed out that the imaging strategy in the CT-arm probably increased the costs by triggering further follow-up visits, hospitalizations, and surgeries.

A financial impact study from the USA collected all SDH cases (acute 14%, subacute 44%, chronic 12%, mixed 30%; *n* = 216) admitted to a tertiary care center between January 2001 and December 2008 [12]. Surgery was performed in 64% of the cases. Median hospital length of stay was 8 days (min–max = 1–99), which was the most important predictor of costs. The median total direct cost for hospitalization was $10,670 (10,820 €). Frontera and colleagues [13] conducted a registry study between 1998 and 2007 showing that the national costs of SDH increased by 60% over the last decade in the USA.

Our costs (mean 5250 € for operatively treated patient) were notably lower than the costs reported previously from Switzerland and the USA. However, the costs are difficult to compare between countries due to differences in case ascertainment, study design, health care systems, and economic issues. The most important predictor of costs has been the length of hospital stay, which was longer in previous studies compared with ours (median 3 days). In our study, the total costs of CSDH increased over time until the 2006–2010 period (50% increase from 1990 to 1995) because of the increase in number of patients treated. However, towards the end of the study period (2011–2015), there was a decline in total costs, despite a greater number of patients treated, in association with a decrease in hospital stay and fewer recurrences. Inpatient time in our neurosurgery clinic decreased during the study period from 5 days (1990–1995) to 3 days (2011–2015) meaning earlier discharge to home or transfer to rehabilitation. The proportion of patients discharged home did not change significantly during the 26-year period. Correspondingly, it can be assumed that the rehabilitation periods after CSDH treatment have become longer towards the end of the study period. In conclusion, while the total direct neurosurgical hospital costs have temporally decreased, there might have been an increase in the rehabilitation costs.

### Strengths and limitations

Our series represents the most extensive non-register-based study of consecutive CSDH cases treated in one neurosurgical department. Although retrospective in nature, the population-based setting makes it less prone to selection bias. Moreover, all the data were collected by one of the authors (M.R.).

This study has several limitations. ICD-codes were used to retrospectively identify all the patients of interest. However, there is a possibility that some cases were not recognized due to incomplete or incorrect ICD-coding. It is likely that all the patients undergoing surgery were identified, but the ICD-coding can be incomplete among the non-operatively treated patients, as the neurosurgeon has only been consulted on these cases. Also, head CT was done only rarely early in the study period; thus, incidental CSDHs were almost never identified. Head CT became widely used in the late 1990s after discovery of thrombolytic agents for ischemic stroke. In addition, the distinction between subacute and chronic SDH is not always obvious, both in relation to time and neuroradiological characteristics. No definition of CSDH is universally accepted [17]. Subacute SDHs were excluded from this study, because this hematoma subtype is considered to represent an entity of its own [3, 11, 20]. Also, definitions used for recurrences differ [6]. Our definition is quite liberal and included second surgeries during the primary treatment.

Moreover, the cost analysis was performed retrospectively and probably underestimates total costs, but this applies to all our study periods equally. The costs are estimates based on the latest costs and do not take into account temporal changes in prices. The costs reported include only the costs in Tampere University Hospital; they do not take into account any costs of treatment outside our hospital. Considering that only half of those among the age group of 60–79 and one-fifth of those 80 years and older could be discharged home, the costs of, for example, rehabilitation and continuing care are noteworthy. Further studies are needed to investigate the actual overall costs on healthcare and society (e.g., rehabilitation, post-CSDH nursing facility dwelling, medication, and social security reimbursements).

## Conclusions

The population-based incidence of CSDH increased markedly during the study period from 1990 to 2015. The number of cases has increased as the CSDH incidence and the aging population is increasing. Nonetheless, in more recent years, the direct hospital costs declined, perhaps due in large part to shortened hospital stays and fewer recurrences related to use of subdural drains. Reducing recurrences is critical for reducing complications and costs. The oldest group of patients, 80 years or older, did not have higher costs than the others, nor did this group have more frequent complications, besides pneumonia. The majority (92%) of recurrences occurred within 60 days. A 2-month follow-up period after CSDH seems sufficient for most, and CT controls are advocated only for symptomatic patients.
